# Reshaping tumor microenvironment by regulating local cytokines expression with a portable smart blue-light controlled device

**DOI:** 10.1038/s42003-024-06566-y

**Published:** 2024-07-29

**Authors:** Hui Rong Wang, Yi Zhang, Yue Jian Mo, Zhan Zhang, Rui Chen, Xi Bin Lu, Wei Huang

**Affiliations:** 1 LiShizhen College of Traditional Chinese Medicine, Huanggang Normal University, Huanggang, Hubei China; 2 Center for Cell and Gene Circuit Design, CAS Key Laboratory of Quantitative Engineering Biology, Shenzhen Institute of Synthetic Biology, Shenzhen Institutes of Advanced Technology, Chinese Academy of Sciences, Shenzhen, Guangdong China; 3 Department of Biology, School of Life Science, Southern University of Science and Technology, Shenzhen, Guangdong China; 4 Department of Biology, Brandeis University, Waltham, MA USA

**Keywords:** Cancer immunotherapy, Cytokines, Optogenetics, Cancer microenvironment

## Abstract

Cytokines have attracted sustained attention due to their multi-functional cellular response in immunotherapy. However, their application was limited to their short half-time, narrow therapeutic window, and undesired side effects. To address this issue, we developed a portable smart blue-light controlled (PSLC) device based on optogenetic technology. By combining this PSLC device with blue-light controlled gene modules, we successfully achieved the targeted regulation of cytokine expression within the tumor microenvironment. To alter the tumor microenvironment of solid tumors, pro-inflammatory cytokines were selected as blue-light controlled molecules. The results show that blue-light effectively regulates the expression of pro-inflammatory cytokines both in vitro and in vivo. This strategy leads to enhanced and activated tumor-infiltrating immune cells, which facilitated to overcome the immunosuppressive microenvironment, resulting in significant tumor shrinkage in tumor-bearing mice. Hence, our study offers a unique strategy for cytokine therapy and a convenient device for animal studies in optogenetic immunotherapy.

## Abstract (web summary)

A study on optogenetics-based cytokine therapy suggests that the immunosuppressive tumor microenvironment can be reversed by beams of blue-light. This would enable control over the timing and location of cytokine expression at tumor sites.

## Introduction

Cytokines are small, soluble secreted proteins released by cells during physiological or pathological activities, with potent immunomodulatory effects in mammals^1^. Although FDA approved two recombinant cytokines, Interferon (IFNA) and Interleukin-2 (IL-2), as cancer immunotherapy drugs, the application of cytokines in immunotherapy still encounters many barriers and limitations, due to their pleiotropy, off-target effects, and short half-life in circulation^1^. Therefore, for cytokines to be successful drug candidates in cancer immunotherapy, targeted release and maintenance of local concentration within tumors are required. In recent years, several approaches have been developed for improving cytokines immunotherapy, such as engineered cytokines (such as IL-2, IL-12, and IL-15) to enhance the affinities and selectivities^2–5^, modified cytokines to prolong half-lives, form bi-functional cytokines with antibody to increase targeted accumulation^6–8^, or engage with immune cells (T cell, natural killer cell) to improve clinical application^9–11^. Despite the advancements made in enhancing the therapeutic potential of cytokines, their effectiveness remains hindered by toxicity and cytokine release syndrome^1,9^, as there are no effective ways to accurately control the dosage, duration, and distribution of cytokines in vivo. However, achieving precise regulation of spatiotemporal distribution of cytokines in animals remains a significant challenge for traditional biology. To address this issue, we here utilized optogenetics, a modern biotechnology that utilizes blue-light (with a peak wavelength of 460 nm) to precisely control the gene expressions or cellular behaviors^12^. By harnessing the benefits of spatiotemporal controllability, no-residue, and non-toxicity, light can be an excellent tool for regulating cytokines expression when combined with light-switchable modules^13^, without triggering unpredictable immunoreactivity.

Optogenetics primarily involves the use of light-sensitive proteins and light control systems. Despite the growing availability of engineered light-sensitive proteins for research, the development of light control systems has been slower due to the complex multidisciplinary design required and the wide range of research targets involved. For example, to assess the efficacy of immunotherapy, animal models that reflect the complexity of the tumor microenvironment are often required^14^. However, there are currently no commercially available optical systems suitable for animal studies in optogenetic immunotherapy. Previous studies have relied on two conventional methods of illumination for conducting animal experiments. The first method involves global illumination, which uses high-intensity near Infrared (NIR) or blue light to illuminate the entire animal^13,15^. Nevertheless, this approach may be deleterious to the well-being of the animals, including their skin and eyes^16,17^. The second method involves local illumination that glued a fixed or freely movable optical fiber to the individual animal skin^13,18–20^. However, this approach may cause restraint stress for animals. Besides, another type of local illumination is a a wireless injectable optoelectronic device or implantable micro-device, designed to be implanted in the target area of a mouse for illumination^21–23^. These wireless devices can avoid animal restraint, but invasive implants may cause tissue damage or unpredictable immune responses, which should be minimized in immunotherapy. Meanwhile, in order to induce the desired immune response, prolonged exposure to light is often required, which can be range from hours to days^15,18–20,24^. Consequently, the optical system must be modified accordingly to ensure compatibility with immunotherapy, to meet the criteria of wireless, non-invasiveness to animals, portable, and capable of long-term illumination.

In this work, we develop a portable smart light-controllable (PSLC) device to achieve spatiotemporally controlled expression of cytokines in freely moving animals, targeting the tumor sites. The key point of this device is real-time tracking of the pre-labeled tumors, which is achieved by calculating the centroids of the tumor site in the captured images through the aid of the computer vision algorithm. The coordinates of centroids are then sent to the gimbal controller to direct the pointing of the laser. Subsequently, the laser illuminated the pre-labeled tumor area under the control of a single-board computer equipped with self-developed interactive tracking software. Through wireless tracking and non-invasive labeling for animals, the optical device avoids inducing the stress and pain derived from animal restraint, implantation, and global illumination. It maintains the normal physiological functions of animals, and that is vital for immunotherapy studies. To validate this system, we inserted selected murine cytokine genes into a light-switchable expression module to establish stably transfected tumor cell lines. Then, using spatiotemporal control of light, we evaluated the efficacy of selected cytokines in inducing antitumor effects with varying expression levels or durations. Our findings demonstrate that light successfully triggered the expression of targeted genes (murine Interferon-gamma and C-X-C motif chemokine ligand 10, *Ifng* and *Cxcl10*) in pre-labeled tumors. Meanwhile, the release and accumulation of *Ifng* and *Cxcl10* further remodeled the tumor microenvironment, by enhancing tumor-infiltrating immune cells (such as CD4+ and CD8 + T cells), and inducing a T cell-inflamed gene expression profile (contained *Ifng*-responsive genes such as *Irf1*, *Gbp*, *Iigp1*, *Igtp*). These effects further stimulated the antitumor response in the tumor microenvironment, enabling the overcoming of the immunosuppressive environment of solid tumors and reduce the tumor burden in animals. Therefore, in this study, a highly spatiotemporally controllable cytokine releasing strategy was developed to improve the antitumor effects of cytokines, by triggering *Ifng*-responsive genes expression profile and recruiting effector immune cells into the tumors in freely moving animals with non-invasive light.

## Results

### Light-controlled expression of cytokines in vitro

Earlier studies revealed that Interferon gamma (IFNG) plays a critical role in the anti-tumor immune response. The extensively diffusion of this cytokine alters the tumor microenvironment through the induction of CXCL9, CXCL10, and CXCL11, which recruit effect immune cells such as natural killer (NK) cells, T cells, monocytes, and others to the tumor site. This process also activates macrophages, allowing them to exert direct anti-tumor effects and upregulates their antigen presentation pathway, thus increasing the tumor sensitivity for cell-mediated cytotoxicity^25–30^. Meanwhile, the chemokine IFNG-inducible protein 10 (IP-10), which is also called CXCL10, is crucial in producing and recruiting activated effector T cells through its cognate CXC chemokine receptor 3 (CXCR3), thereby contributing to an inflammatory microenvironment^27,31,32^. Inspired by these studies on their potential beneficial effects in anti-tumor therapy, we genetically engineered murine mastocytoma line P815 with light-switchable modules (named P815-IFNG) to produce murine IFNG and CXCL10. P815 cell line is used to establish fast-growing solid tumors locally in syngeneic DBA/2 mice, thereby being used extensively by immunologists to understand the complexities of tumor-host immune responses^33^. Subsequently, this engineered P815-IFNG cell line was used to test whether it could improve the immunosuppressive tumor microenvironment with a beam of light.

**Fig. 1:**
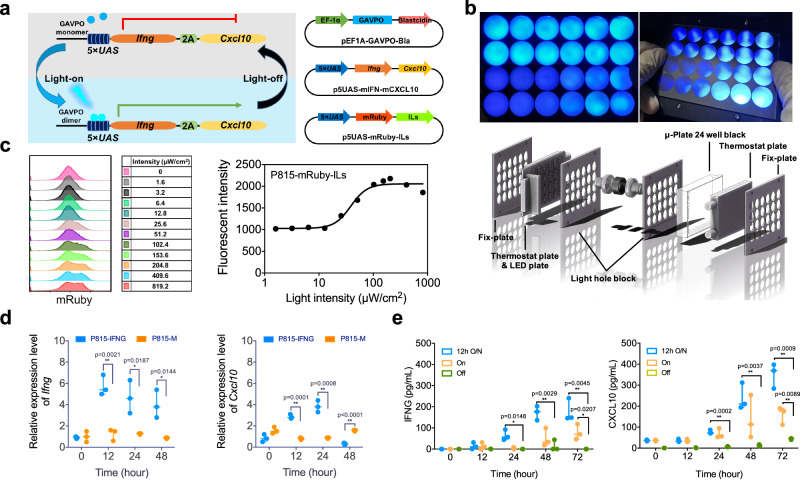
Light-controlled target gene expression in vitro. **a** Schematics of light-controlled gene expression system and plasmids constructions. **b** Regulation of gene expression with light intensities from 0 to 1000 μW cm^-2^ with a customized 24-well plate light-controllable hardware, and **c** the intensity-response curve of mRuby expression in P815-M-ILs cells at 48 h (flow cytometry assay). **d** Time-dependent mRNA expression levels of *Ifng* and *Cxcl10* (qPCR assay, normalized to time zero) in P815-IFNG cells and P815-M cells (control group). **e** Time-dependent production of murine IFNG and CXCL10 proteins (ELISA assay) under the continuous illuminating (On), dark control (Off) and alternative illuminating group (12/12 h light/dark cycles, 12 h O/N). Flow cytometry data is a representative of three independent experiments, each with similar results. Other data are shown as mean ± sem (*n* = 3). All statistical significant were computed against the control group, with *P < 0.05; **P < 0.01.

To identify the ideal light intensity, we initially examined the inducible expression of mRuby (a red fluorescent protein) under the control of blue-light. Specifically, to construct a stable P815-M-ILs cell line, we assembled an EF-1α promoter-driven light-sensitive transactivator GAVPO modules and a 5×UAS promoter-driven mRuby and interleukins, as illustrated in Fig. 1a. These two plasmids were co-transfected into P815 cells. Next, the P815-M-ILs cells were seeded into a 24-well μ-plate and exposed to varying light intensities using a 24-channel light illumination apparatus^34^ (Fig. 1b). As shown in Fig. 1c, the fluorescence intensity of mRuby exhibit a gradual increase in response to light intensities ranging from 10 to 100 μW cm^-2^, reaching a plateau beyond 100 μW cm^-2^, and a subsequent decrease at approximately 400 μW cm^-2^. Next, P815 cells were transfected with both EF-1α-driven GAVPO and 5 × UAS-driven murine *Ifng-Cxcl10* (constructing the P815-IFNG cell line) and subjected to varying durations of illumination, at a constant light intensity of 100 μW cm^-2^. As shown in Fig. 1d, quantitative PCR (qPCR) analysis indicates a significant increase in mRNA expression levels of both *Ifng* and *Cxcl10* in P815-IFNG cells compared to the P815-M cells (control tumor group). This increase was observed between 12 and 24 h after illumination (Fig. 1d). Consequently, both murine IFNG and CXCL10 were found to be significantly released from P815-IFNG cells after 48 h of illumination. Notably, the group that underwent alternating illumination (12 h light/dark cycles, 12 h O/N) demonstrated more than two-fold increase in cytokine release compared to the group that received continuous illumination (ON), under the same light intensity (Fig. 1e). This phenomena may involve a coupling between transcription initiation and histone acetylation with GAPVO that induced by oscillated light^34^. In short, these in vitro studies revealed the potential to induce cytokine release through light, and also specified the optimal conditions required for this process, including the duration and intensity of the light exposure.

### Design and engineering of portable smart blue-light controlled (PSLC) device

**Fig. 2:**
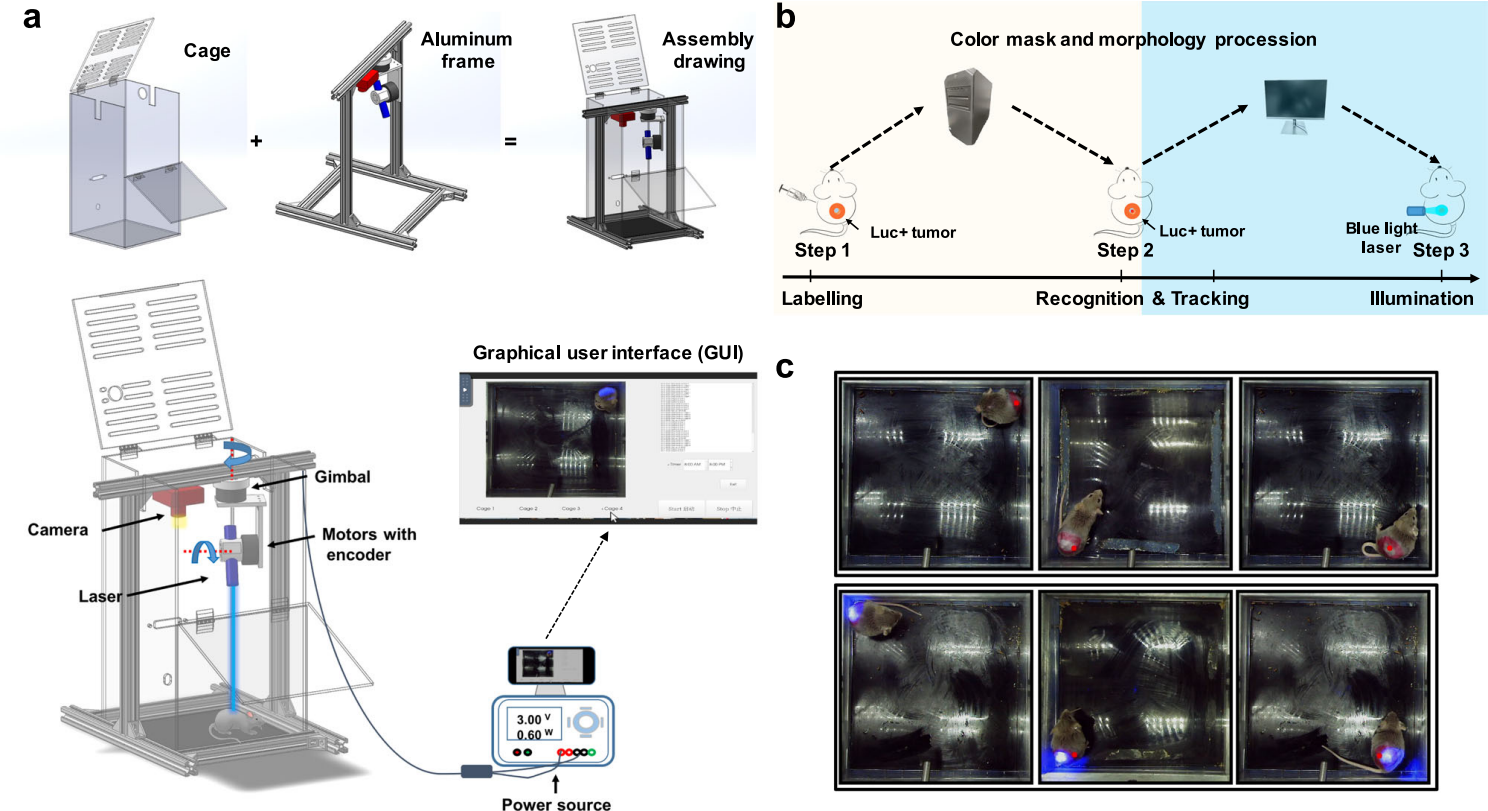
Real-time tracking and illuminating with PSLC device. **a** Assembly drawing of PSLC device, including transparent acrylic cages for illumination, optical tracking hardware, and interactive tracking software that runs on a single-board microcomputer. The entire optical tracking hardware integrates the gimbal with a laser diode module and a camera, which is then mounted on a customized aluminum frame. The tracking software comes with a graphical user interface (GUI) to start programs, set illuminating periods, monitor animal behaviors, and review program logs. **b** The workflow for tumor tracking with PSLC device, includes three steps: labeling, tracking, and illumination. **c** Representative animal images from preliminary testing of the PSLC device. Tumors (within red circle), Red spot (calculated centroid), Blue area (laser illuminating area).

As previously mentioned, to conduct optogenetic immunotherapy studies, the light-controllable system must meet specific requirements such as wireless functionality, non-invasiveness, long-term sustainability, and portability. To fulfill these requirements, we have developed a PSLC device that includes four acrylic cages (with ventilated rails and water bottle snaps), optical tracking hardware (an aluminum gantry with a camera, laser, gimbal, and controller), and interactive tracking software (Fig. 2a). The cages have been designed to provide adequate space for animals to move freely and provide free access to food and water during illumination, ranging from hours to days. To assist researchers with limited coding experience, we have developed a graphical user interface (GUI) that enables them to initiate programs, set illumination periods, monitor animal behaviors, and review program logs on a single-board computer (Fig. 2a). With the help of the optical tracking hardware and software combination, up to four mice can be individually illuminated simultaneously with this device.

In order to enable the tracking and illumination of freely moving mice in real-time, a three-step process was implemented, involving labeling, tracking, and illumination (Fig. 2b). Firstly, the tumor areas of the mice were labeled with an animal marker pen (in the form of a red circle). Subsequently, images of the marked areas were captured using a camera, and a color mask is generated by an algorithm to recognize the labeled col or of the target area, which is used as the input for computer vision tracking process. Next, to deal with environmental noises, the color mask undergoes a morphological process, which includes erosion and dilation. This process generates a simplified and well-defined mask image that aids in recognizing the contour of the target blocks. Then, the centroid of the blocks is calculated frame-by-frame (red dot in Fig. 2c), and this creates a continuous track of the tumor sites of the mice. Finally, the centroid is calculated and converted into coordinates, and sent to the gimbal controller, which guides the laser diode points to the centroid and illuminates the tumor area (blue area in Fig. 2c). The in vivo studies demonstrate that the PSLC device is capable of accurately tracking and illuminating freely moving mice in real-time (Fig. 2c, Supplementary movie 1 and 2). Moreover, the software can be connected to any computer or mobile phone via remote networking, enabling independent control and monitoring of each cage from anywhere and at any time.

### Light-controlled expression of cytokines in vivo

To evaluate whether the PSLC device can be used in animal models for optogenetic immunotherapy, we first determined the optimal light intensity for the in vivo studies.

**Fig. 3:**
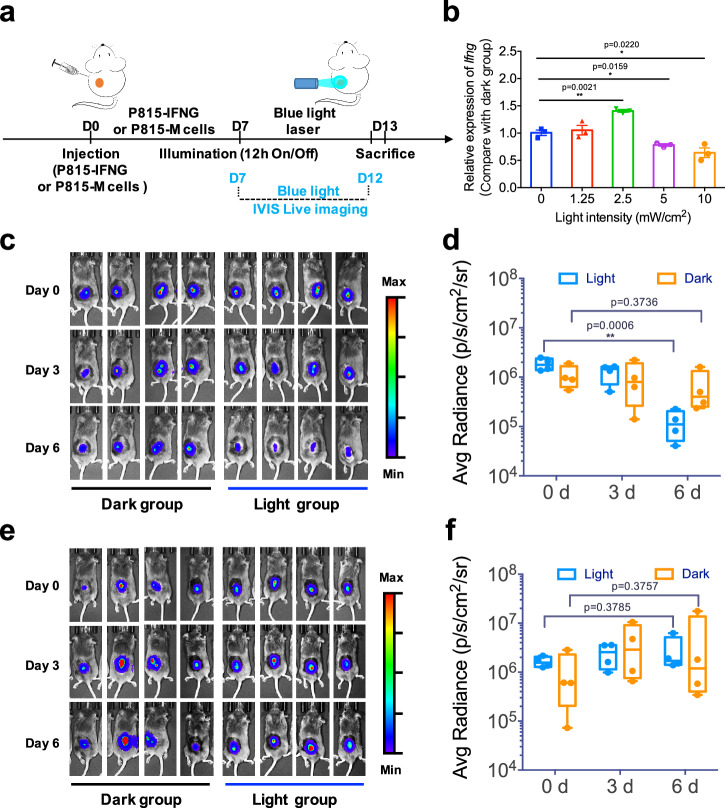
Murine tumor models illuminated by PSLC device. **a** Timeline of in vivo experiment using DBA/2 mice bearing P815-IFNG or P815-M tumors. **b** After 3-day illumination, the relative expression levels of *Ifng* in engineered tumors under different light intensities (0, 1.25, 2.5, 5, 10 mW cm^-2^). The highest expression level of *Ifng* appeared at 2.5 mW cm^-2^. **c** Representative BLI images of P815-IFNG tumor-bearing mice were taken on different days (Day 0, Day 3, and Day 6) after illumination. **d** Tumor size was quantified with average radiance value and compared to initial value at day 0 (the first day of illumination). **e** Representative BLI images of P815-M tumor-bearing mice were taken on different days (Day 0, Day 3, and Day 6) after illumination. **f** Tumor size was quantified with average radiance value and compared to baseline signal at day 0 (the first day of illumination). Data presented as mean ± s.e.m (*n* = 4). Statistic significant was computed against the control group as *P < 0.05; **P < 0.01.

As shown in Fig. 3a, P815-IFNG cells were injected subcutaneously into the back of DBA/2 mice to generate tumor-engineered animal models. Six days later, the tumor area was labeled and exposed to varying light intensities (0, 1.25, 2.5, 5, 10 mW cm^-2^), using the PSLC device (12 h O/N). The animals were sacrificed after 3-day illumination, and tumor tissue was collected to determine the expression of *Ifng*. The results of the quantitative PCR (qPCR) revealed that the intensity of 2.5 mW cm^-2^ of blue light (460 nm peak) led to the highest expression level of *Ifng*, as shown in Fig. 3b. Subsequently, all mice in the light group were subjected to the same light intensity of 2.5 mW cm^-2^. Bioluminescent imaging (BLI) of mice was conducted every three days during illumination. After six days, a significant reduction in the size of engineered tumors (P815-IFNG) was observed in the light group as compared to the dark group of mice (Fig. 3c, d). Then, all the mice were sacrificed for further analysis, and we observed a significant decrease in the number of white blood cells (WBC) and lymphocytes in the light group (Supplementary Fig. 1a, b). In contrast, there are no differences in the number of neutrophils, monocytes, eosinophils, and basophils between the dark and light groups (Supplementary Fig. 1c–f). These findings provide clear evidence that blue-light-induced local cytokine release significantly alters the composition of the immune macroenvironment. In short, by using the PSLC device to track and illuminate the target area of mice in real-time, we not only identified the optimal blue light intensity required for the successful release of target cytokines in animals, but also observed that the localized release of selected cytokines resulted in successful regression of tumors in mice.

To investigate the potential effects of blue light exposure on control tumors in vivo, we established a control tumor model (without the light-controlled cytokine module) using a similar method. Specifically, the control tumor models were produced by injecting P815-M cells subcutaneously into the backs of DBA/2 mice (Fig. 3a). Six days later, the tumor area was labeled and illuminated using the PSLC device (12 h O/N), and BLI of mice was taken every three days during illumination. The findings indicate that, unlike the P815-IFNG engineered tumor model, the size of the control tumor (P815-M) did not exhibit any significant alterations between the light and dark group (Fig. 3e–f). After six days of illumination, all of the mice were euthanized. The blood analysis showed that there were no further changes in the number of white blood cells (WBC) (Supplementary Fig. 2a), lymphocytes (Supplementary Fig. 2b), neutrophils (Supplementary Fig. 2c), monocytes, eosinophils, and basophils (Supplementary Fig. 2d–f) between the two groups. In brief, these findings provide additional evidence supporting the idea that the decrease in tumor size is specifically attributed to the targeted cytokines, rather than the light.

### Application of the PSLC device in optogenetic immunotherapy

**Fig. 4:**
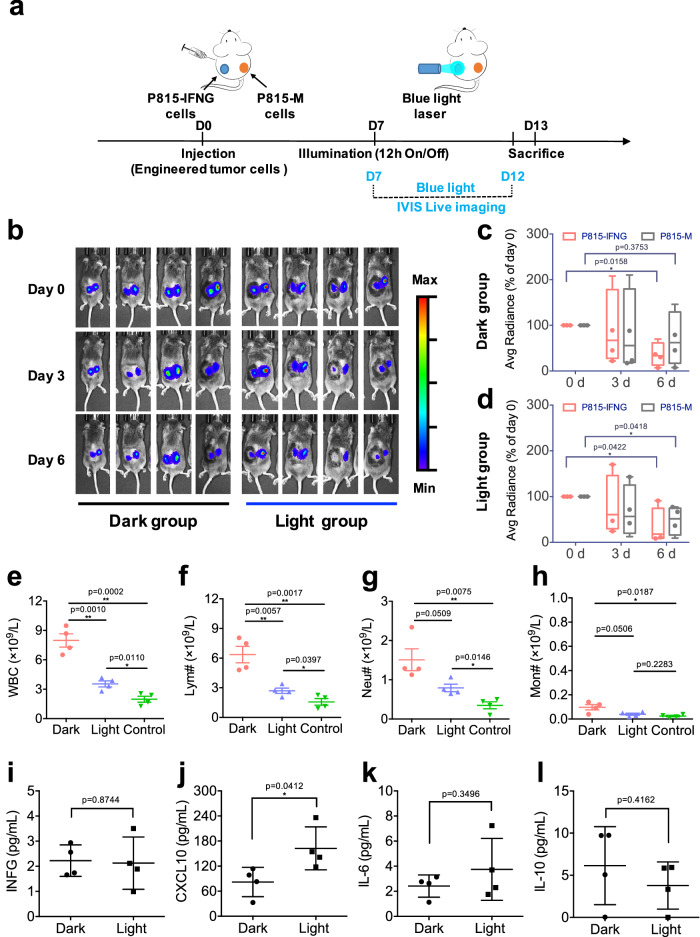
Comparison of control tumor and engineered tumor models under illumination. **a** Timeline of in vivo experiment using DBA/2 mice with matched bilateral tumors (Left: P815-IFNG; Right: P815-M). **b** During illumination, representative BLI images of bilateral tumor-bearing mice at different times (Day 0, Day 3, and Day 6). **c**–**d** Representative BLI images of bilateral tumor-bearing mice were taken on different days (Day 0, Day 3, and Day 6) after illumination. Tumor size was quantified with average radiance value and compared with BLI signal at day 0 (the first day of illumination). **e**–**g** Blood analyses were performed after 6-day illumination, and the results show changes in the number of white blood cells (WBC) **e**, lymphocytes **f**, neutrophils **g**, monocytes (**h**) between light (2.5 mW cm^-2^), dark and control groups. **i**–**l** The concentration of murine INFG **i**, CXCL10 **j**, IL-6 **k**, and IL-10 (**l**) in plasma by the end of day 6 under illumination. Data are shown as mean ± s.e.m (*n* = 4). Statistic significant was computed against the control group as *P < 0.05; **P < 0.01.

Having accomplished both real-time tracking and prolonged illumination of animals using the PSLC device, we observed significant changes in the number of immune cells in tumor-engineered (P815-IFNG) mice in the light group. Based on these results, we postulated that the cytokines released locally could have an impact on the entire immune system. To confirmed this, we subcutaneous injected P815-IFNG cells into the left side and P815-M cells into the right side of DBA/2 mice, to establish bilateral-tumor-bearing mice (Fig. 4a). After six days, the left tumor area (P815-IFNG) was labeled and illuminated with the PSLC device (12 h O/N). BLI of mice was taken every three days during illumination. Seven days later, the animals were sacrificed for further analysis (Fig. 4a). Consistent with previous results, we observed significant tumor regression on the left side (P815-IFNG tumors) in the light group (Fig. 4b, c). Surprisingly, we also observed a lesser extent of tumor reductions on the right side (P815-M tumors) (Fig. 4b, d). It appears that the released cytokines may have an inhibitory effect on adjacent control tumors (Fig. 4b–d).

Subsequently, we analyzed cytokines and blood cells to investigate the intrinsic mechanisms. The blood analysis showed that after 6-day of illumination, the count of WBC, lymphocytes, neutrophils and monocytes decreased significantly, and approached to control levels (Fig. 4e–h), while other immune cells (such as eosinophils, and basophils) had no further changes. These findings provide further evidence that locally released cytokines revert the tumor-disrupted immune macroenvironment by reshaping the composition of systemic immune cells. Furthermore, the cytokines analysis revealed that the expression level of CXCL10 was the only one increased significantly in the light group, (Fig. 4j). Whereas INFG and other cytokines, such as IL-6 and IL-10, did not exhibit any significant increase (Fig. 4i,k, l), this implies that the elevated levels of CXCL10 triggered by light may contribute to tumor regression. Besides, qPCR analysis was performed to determine the expression of target genes in the tumor from both sides. The results show that the expression level of *Ifng* was found to be increased 4-5 times in the left tumor (P815-IFNG) of the light group (Supplementary Fig. 3a), in the right tumor (P815-M), there was no significant difference between the light and dark group (Supplementary Fig. 3b). These findings suggest that the remodeled immune macroenvironment, in turn, affects the tumor microenvironment. In short, these findings illustrate that the light-triggered production of murine IFNG and CXCL10 in the engineered P815-IFNG tumors not only leads to its own reduction, but also causes regression of control tumors (P815-M tumors), potentially by activating a systemic immune response.

### Cytokine-induced changes in the tumor microenvironment

**Fig. 5:**
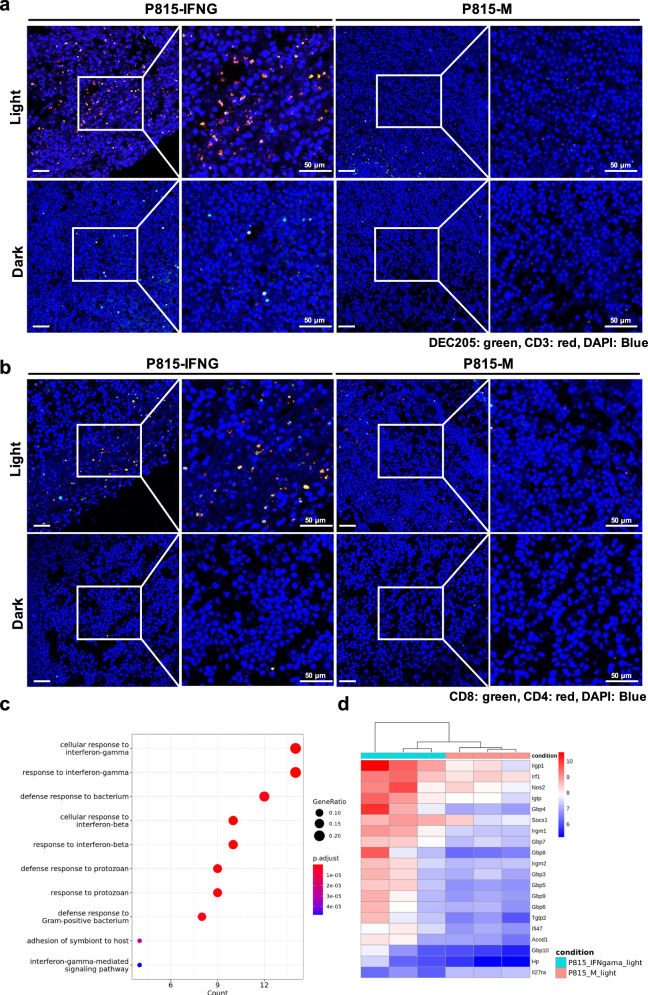
Immunofluorescence (IF) staining and RNA-seq analysis of tumors from single-tumor-bearing mice. After 6-day illumination, tumor tissues were resected from these P815-IFNG or P815-M single-tumor-bearing DBA/2 mice, to analyze the infiltration of immune cells and gene expression profile. **a** Tumor slices were stained with combinations of anti-DEC205 antibody and anti-CD3 antibody, or **b** combinations of anti-CD8 antibody and anti-CD4 antibody. The nuclei of tumor slices were stained with DAPI and imaged using confocal fluorescent microscope with a 100X objective. Representative images from three independent experiments are shown. **c** The GO enrichment analysis of differentially gene sets for various biological processes (qvalueCutoff = 0.05), and **d** the differentially expressed genes between engineered tumors (P815-IFNG) and control tumors (P815-M) in the light group (adjusted *p*-value ≤ 0.05 and the absolute value of the Log2 Fold Change ≥ 1). Representative images from three mice in each group are shown (scale bar = 50 μm).

Based on our prior experiments, to clarify whether the reduction of lymphocytes is due to destruction or tissue infiltration, we conducted immunofluorescence (IF) staining and RNA-seq analysis. The results of IF staining in single-tumor bearing mice showed a marked rise in the infiltration of dendritic cells (DCs) and T cells in P815-IFNG tumor tissues, as compared to the dark group or P815-M tumors (Fig. 5a, b). These findings indicated that the light-induced cytokines effectively increased the number of tumor-infiltrating immune cells. While in the light group of double-tumor bearing mice, similar results were observed in left-side tumors (P815-IFNG). Surprisingly, we also observed a small amount of DCs and T cells in the P815-M tumors (right side) (Supplementary Fig. 4a, b). These tumor-infiltrating immune cells may be responsible for the decrease in lymphocytes in the blood in the light group.

Further confirmation was obtained through RNA-seq analysis, which revealed a high expression level of genes associated with the positive regulation of immune-related molecules (such as *Irf1*, *Acod1*, *Parp9*, *Stat1*, *Irgm*, *Gbp*, *Rsad2*, *Cd226*, *Cxcl10*) in the engineered tumors (P815-IFNG) of the light group (Supplementary Fig. 5a, b), in contrast to those in the dark group. Meanwhile, it was observed that in the light group, the expression of IFNG-related or induced molecules (such as *Irf1*, *Gbp*, *Iigp1*, *Igtp*, *Tgtp2*, *Socs1*, *Irgm*, *Nos2*, and *Ifi47* genes) was increased in engineered tumors (P815-IFNG), rather than P815-M tumors (Fig. 5c, d). In short, the light-induced local release and persistence of IFNG caused remarkable alterations in the tumor microenvironment and triggered tumor regression, primarily by upregulating the expression of immune-related genes and recruiting effect immune cells.

**Fig. 6:**
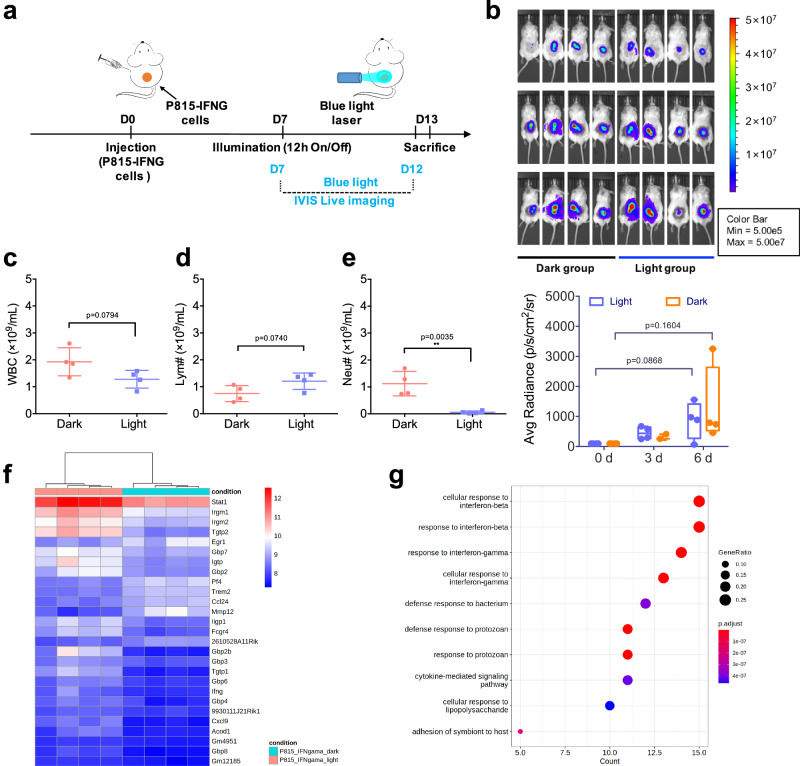
Triple immunodeficient murine tumor models illuminated by PSLC device. **a** Timeline of in vivo experiment using NCG mice bearing P815-IFNG tumors. **b** Representative BLI images of P815-IFNG tumor-bearing NCG mice were taken on different days (Day 0, Day 3, and Day 6) after illumination. Tumor size was quantified with average radiance value and compared with BLI signal at day 0 (the first day of illumination). **c–e** Blood analyses were performed after 6-day illumination, and the results show changes in the number of white blood cells (WBC) (**c**), lymphocytes (**d**), neutrophils (**e**) between light (2.5 mW cm^-2^) and dark groups. **f** The differentially expressed genes between engineered tumors (P815-IFNG) and control tumors (P815-M) in the light group (adjusted *p*-value ≤ 0.05 and the absolute value of the Log2 Fold Change ≥ 1), and **g** the GO enrichment analysis of differentially gene sets for various biological processes (qvalueCutoff = 0.05). Data presented as mean ± s.e.m (*n* = 4). Statistic significant was computed against the dark group or control group (Day 0) as *P < 0.05; **P < 0.01.

Subsequently, we chose the NOD/ShiLtJGpt-Prkdc^em26Cd52^Il2rg^em26Cd22^/Gpt (NCG) mice to validate this conclusion. These triple immunodeficient mice lack mature and functional T cells, B cells, NK cells, and exhibit impaired macrophage and DCs function, making them an ideal animal model for immuno-oncology studies. According to Fig. 6, after 6-day illumination (Fig. 6a), the engineered tumors (P815-IFNG) showed a notable increase in both the light and dark groups (Fig. 6b), with no significant differences between the two groups. Additionally, the blood analysis indicated that light had a significant impact on reducing the number of neutrophils (Fig. 6e), but did not affect the count of WBC and lymphocytes (Fig. 6c, d). These results indicated neutrophils may involve in tumor progression in NCG mouse, as confirmed by RNA-seq analysis (Fig. 6f, g). Specifically, two genes (*Fcgr4 * and *Acod1* ) that are associated with neutrophil activation and antimicrobial response of innate immune cells were found to be upregulated in the tumors (P815-IFNG) of the light group.

Additionally, despite observing a high level of gene expression associated with the positive regulation of immune-related molecules (such as *Stat1*, *Irgm*, *Gbp*, *Cxcl9*), and IFNG-related molecules (such as *Gbp*, *Igtp*, *Ligp1*, *Tgtp1* genes) in the light group, tumor growth was not inhibited due to the absence of effective immune cells in NCG mice. Therefore, by utilizing the triple immunodeficient NCG mice, we have further confirmed that the local released cytokines by light primarily elicit their anti-tumor effects by attracting and activating effect immune cells (such as T cells), which is the key step in light-controlled cytokines therapy in solid tumors.

## Discussion

As powerful small immunomodulatory proteins, the application of cytokines in cancer immunotherapy is limited by their pleiotropy, narrow therapeutic window, and short half-life in circulation^1^. Despite attempts by scientists to overcome these limitations, there are still barriers to effectively control the local expression of cytokines and induce sustained cellular responses in animals. However, the spatiotemporal control of gene expression is difficult to achieve with traditional biotechnologies due to the lack of appropriate tools. Fortunately, the rapid development of multidisciplinary optogenetics offers opportunities. It enables precise control of gene expression in space and time through highly tunable light. Based on this technique, we designed a PSLC device that enables wireless and non-invasive illumination, thus preventing animal restraint stress and unpredictable immune responses during experiments^35,36^.

To achieve targeted induction of cytokines in freely moving animals, the key step is real-time tracking. Here, we place a pre-labeled mouse in an acrylic cage (Supplementary Fig. 6a), and designate the whole black bottom of the cage as the field of view for the camera. Subsequently, animal images were captured and instantly transmitted to the computer for real-time display in the monitoring window. Furthermore, a self-developed interactive tracking program was utilized to morphologically process the images and generate animal moving trajectories, which were composed of the centroids of the target areas (Fig. 2c. red dot: calculated centroid). Next, the transformed centroid coordinates were promptly transmitted to the gimbal controller for laser pointing guidance. Ultimately, by fine-tuning the x, y, and z axis parameters, the targeted area could be accurately identified and illuminated. In brief, based on OpenCV, an open-source package in computer vision (CV), we developed an interactive tracking software for freely moving animals. This software is a handy tool for researchers with limited coding experience, as it realizes real-time tracking and displaying camera visions by simply turning on the software. The entire PSLC device was placed on a removable double-layer steel shelf, including optical tracking hardware, two power supplies, and a single-board computer (Supplementary Fig. 6b). The entire device can be sterilized with a UV lamp to maintain specific pathogen free (SPF) living conditions for animals. In conclusion, the optical tracking hardware and software of the PSLC device ensure targeted illumination of the animals, avoid restraint stress and implantation pain on the animals, and maintain their normal physiological functions during the experiment. At the same time, the software can be connected to control and monitor each cage independently anywhere via remote networking.

As we all know, not all cancer patients can benefit from immunotherapy, mainly due to immunotherapy resistance (or immune-cold tumors)^37,38^. Studies revealed that the major predictive factor of these ‘cold’ tumors is the lack of sufficient T cell infiltration and a T cell-inflamed gene expression profile in tumors^39,40^. Therefore, recruiting effector T cells into the tumor environment is a critical step for immunotherapy. Based on this and the PSLC device, we proposed constructing a light-controlled cytokine release system to enhance tumor-infiltrating immune cells. According to previous studies, the chemokine CXCL10 plays a predominant role in trafficking activated T lymphocytes (CD4 + T cells and CD8 + T cells) to the site of inflammation, through binding to its corresponding receptor CXCR3^31,41^. Elevated levels of CXCL10 were associated with tumor-infiltrating CXCR3+ immune cells, including activated T cells, macrophages, DCs, monocytes, and NK cells. That coincided with reduced tumor growth^41–43^. Subsequently, these activated immune cells produced IFNG, a cytokine that is categorized as a tissue modifier and highly correlated with the T cell-inflamed tumor microenvironment^44,45^. Moreover, the released IFNG in turn activates dendritic cells and macrophages to produce chemokines such as CXCL9 and CXCL10 to recruit additional CXCR3+ immune cells^44^. Based on these findings, we designed a light-controlled *Infg*-*Cxcl10* gene loop to manipulate the tumor microenvironment. Here, our results show that the positive feedback loop not only contributes to overcoming the immunosuppressive tumor microenvironment but also alters the composition of the immune macroenvironment. This, in turn, affects the tumor microenvironment and ultimately reduces tumor burden in animals.

Considering that long-term exposure to IFNG alters tumor phenotypes and a dose-dependent effect of IFNG in tumor microenvironment^29,46^, the *Ifng-Cxcl10* loop needs to be precisely regulated to avoid producing side effects. Using the PSLC device, we first achieved concentration- and time-dependent expression of target cytokines within tumors by light. However, considering that high-dose cytokines (e.g., IL-2, IL-12, TNF, and IFNG) treatments may cause cytokine storm, subsequently induce severe side effects and multi-organ dysfunction in animal models^47–49^. We here measured levels of several cytokines (e.g., IFNG, CXCL10, IL-6, and IL-10) that may associate with target cytokines or cytokine storm^50^, as well as circulating white blood cells (WBC) in animals that may be affected by the light or target cytokines. The results of the P815-IFNG tumor models displayed a significant increase in the transcriptional (Supplementary Fig. 3a) or translational (Fig. 4j) levels of murine IFNG and CXCL10 in the light group, rather than IL-6 or IL-10 (Fig. 4k, l). In addition, locally released CXCL10 recruits enhanced tumor-infiltrating immune cells in the tumor microenvironment (Fig. 5a, b), while sustained induction of IFNG leads to a T cell-inflamed gene expression profile in tumors^44^ (Fig. 5c, d). The results confirmed that this strategy of using light to control local cytokine expression levels effectively transforms “cold” tumors into “hot” tumors. Notably, the light group exhibited a reduction in tumor burden in animals while not inducing detectable autoimmunity, highlighting the convenient controllability of this system.

In conclusion, the utilization of this PSLC device allows us to determine the roles of cytokines and their synergistic effects in immunotherapy, thereby providing valuable evidence to support the clinical application of cytokine therapy. It is therefore a realistic expectation that light-based treatment could be used to treat tumors or other illnesses in the future. Nonetheless, despite the fact that we achieved the concept of using a beam of light to treat solid tumors in animal models, there are still some issues that need to be further addressed, such as the effects of different cytokines combinations, the tracking procedures for tumors at different locations, the definite exposure time of light and the improvement of PSLC device.

## Methods

### Plasmids construction

The lightON system includes two plasmids, A and B. Plasmid A is a light-sensing transcriptional activator GAVPO that contained a GAL4 DNA binding domain, a light-sensing VVD domain a p65 AD activation domain. Plasmid B is a lightON promoter (5×UAS) driven target genes (Fig. 1a). The original plasmids were gifts from Prof. Yi Yang^13^. The mRuby3 plasmid was a gift from Prof. Jun Chu. For light intensity, exposure time studies, and in vitro cytokines release assay, the light-induced transcriptional activator plasmid A1 (PB5- EF-1α -GAVPO-2A-Bla-PB3) was constructed by sub-cloning EF-1α promoter, GAVPO and blasticidin resistance gene (Bla) in a PiggyBac transposon backbone^51^. In contrast, the lightON plasmid B1 (PB5-5×UAS-mRuby-2A-ILs-PB3) was constructed by assembling 5×UAS, mRuby3, and selected interleukins (ILs) with another PiggyBac backbone (Fig. 1a). For in vivo studies, plasmid B2 (PB5-5×UAS-*Ifng* -2A- *Cxcl10*-hGluc-PB3) was constructed by sub-cloning murine *Ifng*, *Cxcl10*, and humanized form of Gaussia luciferase (hGluc)^52^ into PiggyBac backbone (Fig. 1a). Besides, the control plasmid C (PB5- EF-1α -mRuby-2A-Fluc-PB3) was constructed by sub-cloning mRuby3 and firefly luciferase (Fluc) into a PiggyBac backbone plasmid.

### Engineered cell lines

The murine mastocytoma cell line P815 was obtained from the Type Culture Collection of the Chinese Academy of Sciences, and performed mycoplasma detection and cell line authentication. P815 cells were maintained in Dulbecco’s modified Eagle’s medium (DMEM, Thermo Fisher Scientific) supplemented with 10% fetal bovine serum (FBS, HyClone,) at 37 °C under 5% CO_2_. In the illuminating experiments, stably transfected P815 cell with GAVPO and lightON-mRuby-ILs was generated for in vitro studies. Specifically, P815 cells were co-transfected with 450 ng plasmid A1, 450 ng plasmid B1, and 100 ng PGK-transposase plasmids. After 24 h, the cells were further selected with 10 μg mL^-1^ blasticidin (Thermo Fisher Scientific) for another 3 days. Subsequently, these cells were illuminated with blue light (100 μW cm^-2^) for 24 hours, and single-cell clones that only expressing mRuby were sorted into 96-well plates by BD FACSAria SORP. Finally, the single-cell clone with higher levels of mRuby and normal growth ratio was selected and named P815-M-ILs. For in vivo studies, stably transfected P815 cells with GAVPO, lightON-*Ifng*-*Cxcl10*-hGluc and mRuby-Fluc were generated, by co-transfection with 300 ng plasmid A1, 300 ng plasmid B2, 300 ng plasmid C, and 100 ng PGK-transposase plasmids. Similarly, single-cell clones were sorted into 96-well plates and cultured for another 7 days. Before detecting the hGluc activity, each single-cell clone was illuminated with blue light for 24 hours. Ultimately, one single-cell clone, named P815-IFNG was selected for its higher hGluc activity. Besides, as a control cell line, stably transfected P815 cells with mRuby-Fluc were generated with co-transfected with 300 ng plasmid C and 100 ng PGK-transposase plasmids, and the single-cell clone that has a normal growth ratio and expressing mRuby protein was selected, named P815-M. These cell lines were also performed for mycoplasma detection in our laboratory.

### Mice

Animal protocols and procedures were approved by the Laboratory Animal Welfare and Ethics Committee, Southern University of Science and Technology (approval number: SUSTC-2019-104). We have complied with all relevant ethical regulations for animal use. Both male and female DBA/2 mice (6-8 weeks old, specific-free) were purchased from Charles River (Beijing, China), whereas NCG mice of the same age were obtained from GemPharmatech (Guangdong, China). After arrival, animals were maintained in our laboratory animal center to acclimatize for two weeks prior to experimentation. These animals had free access to food and water at a constant temperature (20 ± 1°) and a 12/12 h light/dark cycle.

### In vitro illumination experiment

P815-M-ILs cells (5 × 10^4^ cells per well) were seeded in 24-well μ-plate (ibidi GmbH, Germany). After 24 h, the plate was placed in a 24-channel illumination apparatus (Fig. 1b) with a water cooling pump to ensure cells were cultured at 37 °C. For light intensity studies, a light intensities file (range from 0 to 1000 μW cm^-2^) for 24 wells was generated from MATLAB software (MathWorks, USA), and then loaded into the custom Python code to control the apparatus for 48 hours. While for the duration of light studies, an illuminating time file (range from 0 to 60 h) was generated and used to control the apparatus under the optimal intensity.

### Flow cytometry assay

After 48 h illumination, the stably-transfected P815-mRuby-ILs cells were collected and filtered through a 40 μm cell strainer (BD Falcon) to remove clumps, and further analyzed with a Beckman Cytoflex S cytometer (Brea, US). To ensure the reproducibility of the measurements, we always use fluorescent calibration beads (Sphero Rainbow calibration beads 6 peaks; Catalog no. RCP-60-5; Spherotech) to adjust the instrumentation parameters. After that, the flow cytometry data were analyzed with FlowJo V10 software (Ashland, US) with the gating shown in Supplementary Fig. 7. The mean fluorescent intensity of light inducible reporter (red fluorescent protein, mRuby) in P815-mRuby-ILs cells was calculated.

### Quantitative reverse transcription PCR (RT-qPCR) analysis

Total RNA was extracted from cells at designed time points (0, 12, 24, and 48 h after illumination), or homogenized tumor tissues from mice (after 3 or 6 days of illumination) with Rneasy Mini Kit (Qiagen, Germany) following the manufacturer’s instructions. cDNA was generated by reverse transcription (PremixScript™ RT Master Mix, RR036A, TAKARA) with oligo (dT) primers, with the same amount of template RNA. Quantitative RCR (qPCR) was performed with SYBR ® Premix Ex Taq™ II (RR820A, TAKARA) and a CFX96 Touch Real-Time PCR Detection System (Bio-Rad, USA). The mRNA expression levels of glyceraldehyde-3-phosphate dehydrogenase (GAPDH) were used as an internal control for target genes, and the ^ΔΔ^C_t_ method was used to normalize and calculate fold changes of mRNA. Primers are listed in Supplementary Table 1.

### Cytokines and chemokines analysis

Cell supernatants from in vitro studies were collected at designed time points (0, 12, 24, 48, and 72 h after illumination) to quantify cytokines and chemokines with murine CXCL10 and IFNG ELISA kit (BOSTER, China). Meanwhile, plasma samples from in vivo studies were collected after 6-day illumination, and measured with LEGENDplex™ mouse anti-virus response panel (Biolegend, USA) following the manufacturer’s instructions.

### Immunofluorescence (IF)

Tumor tissues were resected from mice and then fixed with 4% paraformaldehyde (PFA, P1110, Solarbio) at 4 °C overnight. Subsequently, tumor tissues were dehydrated in 15–30% sucrose solution for 3 days, and immersed in O.C.T (Sakura Finetek) for snap-frozen. Then, these samples were cryosectioned at 8 μm with Cryostat (CryoStar NX50, Thermo Fisher Scientific). After antigen retrieval with Quick Antigen Retrieval Solution for Cell Section (C1035, Solarbio), slides were blocking with 5% murine serum for 2 h at RT, then incubated with different primary antibodies (1:300) in blocked solution at 4 °C overnight, including anti-DEC205 antibody (138201, BioLegend), anti-CD3 antibody (clone 17A2, 70-0032-U100, TONBO biosciences), anti-CD4 antibody (clone GK 1.5, 14-0041-82, Thermo Fisher Scientific), anti-CD8α antibody (clone 53-6.7, 14-0081-82, Thermo Fisher Scientific)^53^. After that, slides were washed with PBS, and continued to incubate with different secondary antibodies for 1 h at RT, including Alexa Flour ®488 mouse anti-Rat IgG 2a (1:1200, clone 2 A 8F4, ab172332, Abcam) and Alexa Flour ®647 anti-Rat IgG 2b (1:500, clone 2B 10A2, ab172335, Abcam). Then, the nucleus was stained with DAPI (Thermo Fisher Scientific) for 5 min before confocal imaging. Microscopic analysis for IF staining samples were conducted with Nikon Confocal A1R with FLIM microscope (A1R+Symp64, 20X dry or100X oil objectives), and each fluorescent dye was analyzed with Imaris Viewer (Bitplane, Switzerland).

### Design and engineering of portable smart blue-light controlled (PSLC) device

The PSLC device comprises two programmable DC power supplies (DP832, RIGOL, China), acrylic illumination cage (20 × 20 × 35 cm, with ventilated rails and water bottle snap), optical tracing hardware, and control software with a graphical user interface (GUI) that was running on a single-board computer (Fig. 2a). As shown in Fig. 2a, the transparent acrylic illumination cage (20 × 20 × 35 cm) was designed and custom-made (Oudike, China) for freely moving animals, and equipped with ventilated rails and water bottle snaps. For optical tracing hardware, we first built a 2-axis gimbal that included a brushless gimbal motor (HT3505, HAITAI) with a magnetic encoder (AS5048A, HAITAI). The gimbal was subsequently equipped with a laser diode (HSBD22-450AD100, HS, China), and a gimbal controller (simpleBGC 32-bit, BASECAM) with an inertial measurement unit (IMU). Then, the entire gimbal platform and camera (RER-USBFHD06H-LS36, RERVISION, China) were fixed on the customized gantry-like aluminum frame (21 × 21 × 33 cm). Specifically, the gimbal controller and motors were connected with power lines and encoder data cables. The calibration in 6 directions of IMU was performed to let the accelerometer find the gravity direction, so that the gimbal controller could level the pitch axis automatically. While for the yaw axis, we overcame the yaw drift caused by lack of reference, by setting the original angle parallel to the aluminum frame on the magnetic encoder in the motor. To connect computer vision recognition of mouse position to gimbal angel, the x-y position data was converted into 3-dimensional polar coordinates through trigonometric function, and calibrated with a red rubber cap (keyboard switch cap). The laser can point to where the red marker is located with minimum delay by setting these parameters. Then, mice were labeled and placed in the cages for further testing, and parameters were adjusted to optimize the tracking of animals. In addition, the control panel ran a proportion-integral-derivative (PID) auto-tune program for the brushless motors to keep self-stabilizing during the operating state.

To facilitate researchers with limited coding experience to use the PSLC device, a control software with a graphical user interface (GUI) was developed to start programs, set illuminating periods, monitor animal behaviors, and review program logs on a single-board computer. Meanwhile, any computer or mobile phone can connect it for real-time monitoring through remote networking.

### In vivo illumination experiment

DBA/2 mice (8–10 weeks, 20–25 g in weight) were used in illumination experiments. One million engineered P815-IFNG or P815-M cells were collected, washed, and suspended in 100 μL PBS, and subsequently implanted subcutaneously into the backs of DBA/2 mice (9–10 weeks) (Day 0). Seven days later (Day 7), all experimental mice (*n* = 8) were randomly assigned to groups that were illuminated with (light group, *n* = 4) or without blue light (dark group; *n* = 4). Subsequently, the mice in the light group were housed individually in light-controlled cages (20 × 20 × 35 cm, Fig. 2a) with free water and food supply. Every three days, these cages were cleaned, and all experimental mice were used for in vivo bioluminescence imaging. After another 7 days (Day 14), all the mice were sacrificed, the blood samples were collected for whole blood analysis and plasma separation, while the tumor tissues were removed for RNA extraction.

### In vivo imaging

In vivo bioluminescent imaging (BLI) of live animals was carried out with the IVIS® Spectrum Imaging System (PerkinElmer). Mice were anesthetized by inhalation of a mixture gas (isoflurane and oxygen) via XGI-8 Gas Anesthesia System. Before imaging, D-Luciferin (YEASEN, 3 mg per mouse) was injected intraperitoneally into each mouse, and the bioluminescence signals were obtained after 15 minutes. The signal intensity of the interest region was analyzed with Living Image software, and each image data was normalized and displayed based on the color intensity.

### RNA-seq analysis

The removed tumor tissues were frozen with liquid nitrogen as soon as possible. Total RNA was extracted, quantified, and sequenced on an Illumina HiSeq X Ten platform in OE Biotech Co., Ltd. (Shanghai, China). For RNA sequencing preprocessing, the sequences and annotations of the knock-in genes were added into the mouse mm10 reference genome to build the reference genome for engineered murine tumor cells. Raw FASTQ sequencing reads were filtered and corrected by the Fastp (v0.20.1) with default parameters to get the clean reads and quality reports. Clean reads were splice-aware aligned to the reference genome using STAR (v2.7.9a) and counted using HTSeq (v0.13.5) with default parameters. The counts of the mitochondrial genes in all samples were removed. The ComBat-seq tool in the sva R package (v3.40.0) was used to adjust the batch effect in the counts.

For differential gene expression and enrichment analysis, the genes with a total count <10 reads were removed prior to identifying differentially expressed genes using DESeq2 R package (v1.32.0) and IHW R package (v1.20.0) with default parameters. Genes are considered significantly differentially expressed if the Benjamini-Hochberg adjusted p-value ≤0.05 and the absolute value of the Log2 Fold Change ≥1. The differentially expressed genes between the P815-IFNG and P815-M in the light group were filtered with the genes that were also identified differentially in the dark group. Moreover, the differentially expressed genes in P815-IFNG tumors between the light and dark groups were filtered with the genes that were also identified differentially in P815-M tumors between the light and dark groups. The GO Enrichment Analysis of the filtered differentially gene sets for the biological process was conducted using clusterProfiler R package (v4.0.5) with the qvalueCutoff = 0.05.

### Statistics and reproducibility

The measurements were taken from distinct samples, and the sample sizes were described in each figure legend. Data are presented as mean ± s.e.m. Statistical analysis was performed using Student’s unpaired t-test (two-tailed) by GraphPad Prism (version 7), and a *p*-value < 0.05 was considered significant.

### Declaration of generative AI and AI-assisted technologies in the writing process

During the preparation of this work the authors used ChatGPT in order to improve readability and language. After using this tool, the authors reviewed and edited the content as needed and take full responsibility for the content of the publication.

### Reporting summary

Further information on research design is available in the Nature Portfolio Reporting Summary linked to this article.

### Supplementary information

Supplementary information

Description of Additional Supplementary Files

Supplementary Data 1

Supplementary Data 2.

Supplementary Movie 1 (Tracking)

Supplementary Movie 2 (Illuminating)

Lasing Reporting Summary

Reporting summary

## Data Availability

The source data for main graphs can be found in Supplementary Data 1, while the source data for supplementary information can be found in Supplementary Data 2. The raw data of RNA-seq and processed data files have been deposited in the NCBI GEO under the accession-number GSE214760, and the analysis code at https://github.com/QBioLab/Cancer-treatment-project-hui-rong. Other data, such as the real-time tracking and analytical datasets that are too large to be publicly shared, yet they are available from the corresponding author, upon reasonable request.

## Code availability

The positioning for the entire gimbal platform in the PSLC device is controlled by a customized python code, using a serial command that can be found at GitHub: https://github.com/QBioLab/let-there-be-light.
