# Supplementary material for: Reshaping tumor microenvironment by regulating local cytokines expression with a portable smart blue-light controlled device
Source: Commun Biol. 2024 Jul 29;7:916. doi: 10.1038/s42003-024-06566-y (PMC11289142; doi:10.1038/s42003-024-06566-y)
Supplement: Supplementary file 1 — Supplementary information [file 42003_2024_6566_MOESM1_ESM.pdf]

Reshaping tumor microenvironment by regulating local cytokines expression with a portable smart blue-light controlled device

Hui Rong Wang[a,c *], Yi Zhang[d], Yue Jian Mo[b,c],

Zhan Zhang[c], Rui Chen[c], Xi Bin Lu[c], Wei Huang[b,c *]

a. LiShizhen College of Traditional Chinese Medicine, Huanggang Normal University, Huanggang, 438000, Hubei, China.

b. Center for Cell and Gene Circuit Design, CAS Key Laboratory of Quantitative Engineering Biology, Shenzhen Institute of Synthetic Biology, Shenzhen Institutes of Advanced Technology, Chinese Academy of Sciences, Shenzhen, 518055, Guangdong, China.

c. Department of Biology, School of Life Science, Southern University of Science and Technology, Shenzhen, 518055, Guangdong, China.

d. Department of Biology, Brandeis University, Waltham, 02453, Massachusetts, US.

Supplementary Figures

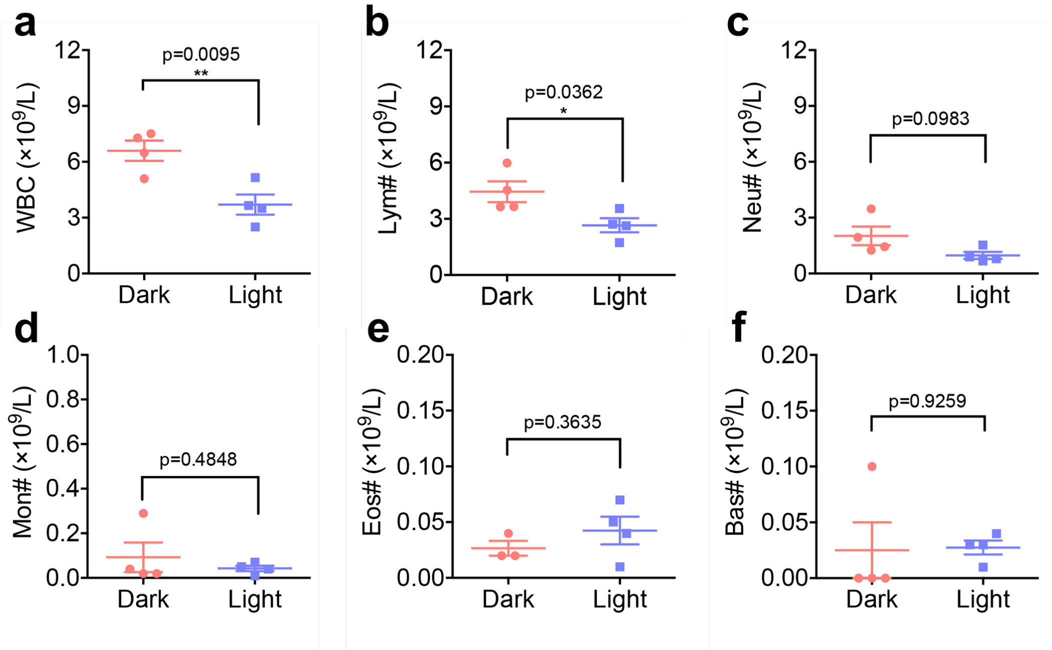

Supplementary Figure 1. Blood analyses of engineered (P815-IFNG) mouse tumor models illuminated by PSLC device. Blood analyses were performed after 6-day illumination, and the results show changes in the number of white blood cells (WBC) (**a**), lymphocytes (**b**), neutrophils (**c**), monocytes (**d**), eosinophils (**e**), and basophils (**f**) between light (2.5 mW cm^{-2}) and dark groups. Data are shown as mean ± s.e.m (n=4). Statistic significant was computed against the control group with * $P < 0.05$, ** $P < 0.01$.

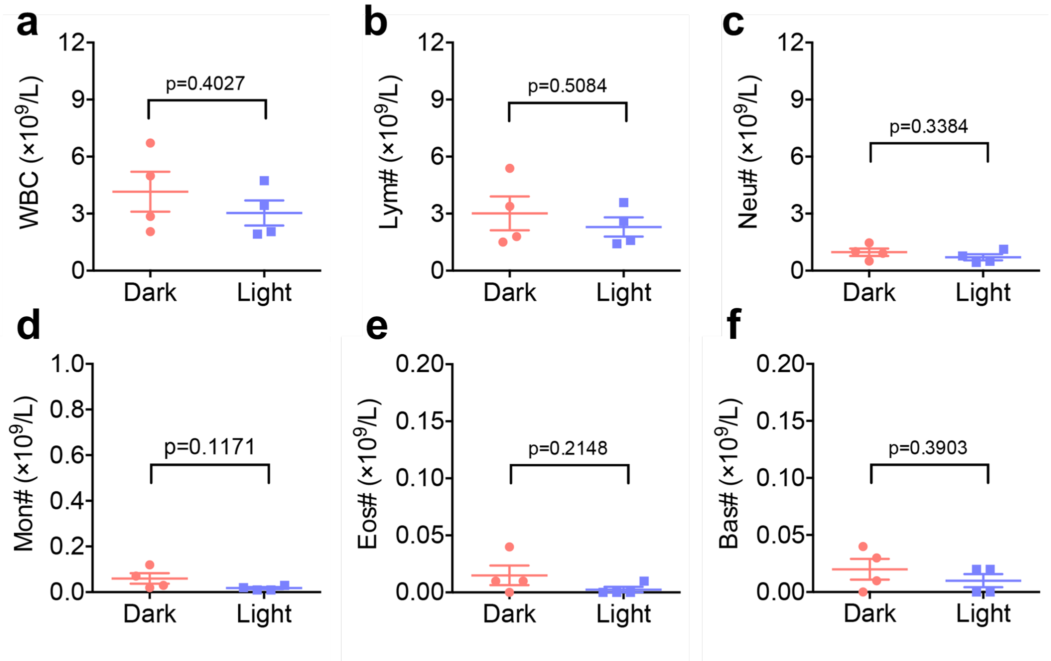

Supplementary Figure 2. Blood analyses of normal (P815-M) mouse tumor models illuminated by PSLC device. Blood analyses were performed after 6-day illumination, and the results show changes in the number of white blood cells (WBC) (**a**), lymphocytes (**b**), neutrophils (**c**), monocytes (**d**), eosinophils (**e**), and basophils (**f**) between light (2.5 mW cm^{-2}) and dark groups. Data are shown as mean ± s.e.m (n=4). Statistic significant was computed against the control group.

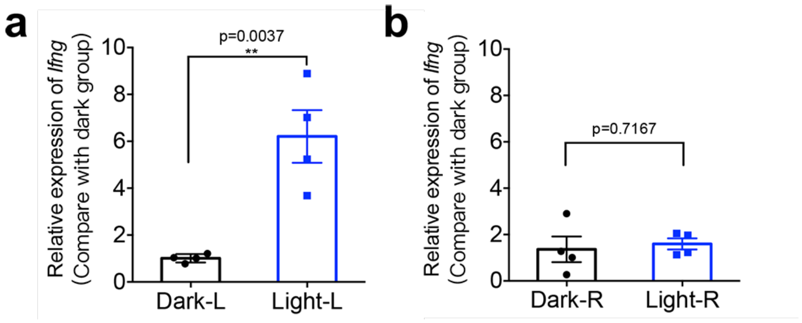

Supplementary Figure 3. The relative mRNA expression levels of *Ifng*. After 6-day illumination, tumor tissues were resected from these bilateral-tumor-bearing DBA/2 mice, with P815-IFNG tumors in the left (L) and P815-M tumors in the right (R), to analyze the mRNA expression levels of *Ifng*. The mRNA expression levels of *Ifng* in P815-IFNG tumors (**a**), and P815-M tumors (**b**) between the light and dark group(n=4), with * $P < 0.05$, ** $P < 0.01$.

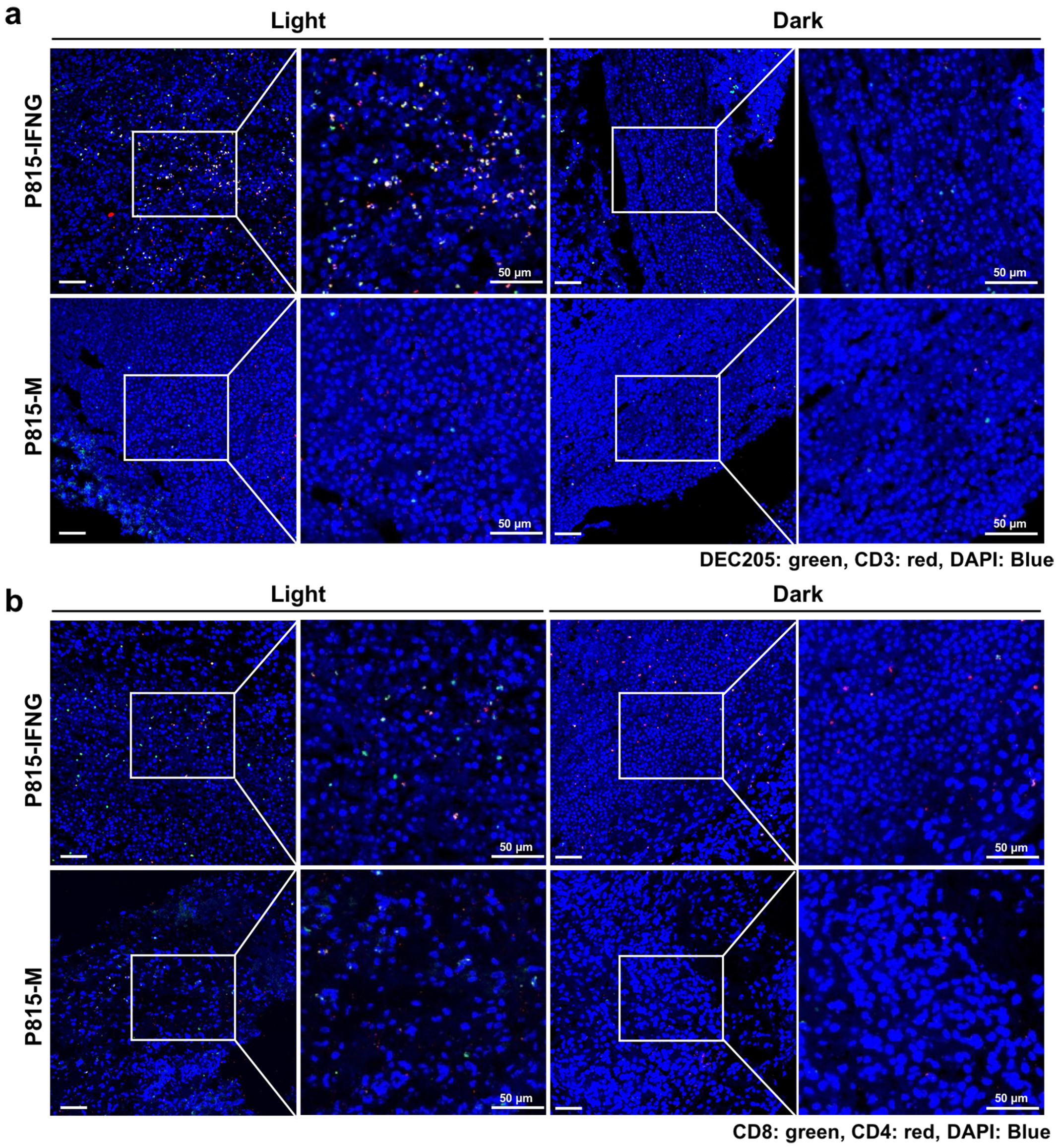

Supplementary Figure 4. Immunofluorescence (IF) staining of tumors from bilateral-tumor-bearing mice.

After 6-day illumination, tumor tissues were resected from these P815-IFNG and P815-M bilateral-tumor-bearing DBA/2 mice, to analyze the infiltration of T cells and DCs. **a**. Tumor slices were stained with combinations of anti-DEC205 antibody and anti-CD3 antibody, or combinations of anti-CD8 antibody and anti-CD4 antibody (**b**). The nuclei of tumor slices were stained with DAPI and imaged using confocal fluorescent microscope with a 100X objective. Representative images from three independent experiments are shown (scar bar = 50µm).

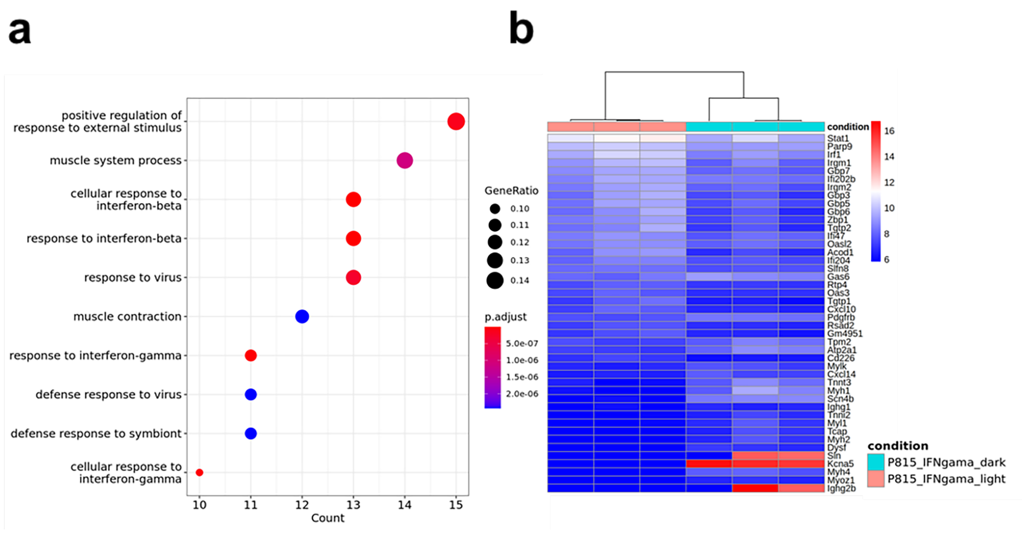

Supplementary Figure 5. RNA-seq analysis of tumors from single-tumor-bearing mice.
After 6-day illumination, tumor tissues were resected from single-tumor-bearing DBA/2 mice (P815-IFNG) to analyze the gene expression profile. **a**. The GO enrichment analysis of differentially gene sets for various biological processes (qvalueCutoff = 0.05), and (**b**) the differentially expressed genes in engineered tumors (P815-IFNG) between the dark group and light groups (adjusted p-value ≤ 0.05 and the absolute value of the Log2 Fold Change ≥ 1). Representative images from three mice in each group (n=3) are shown.

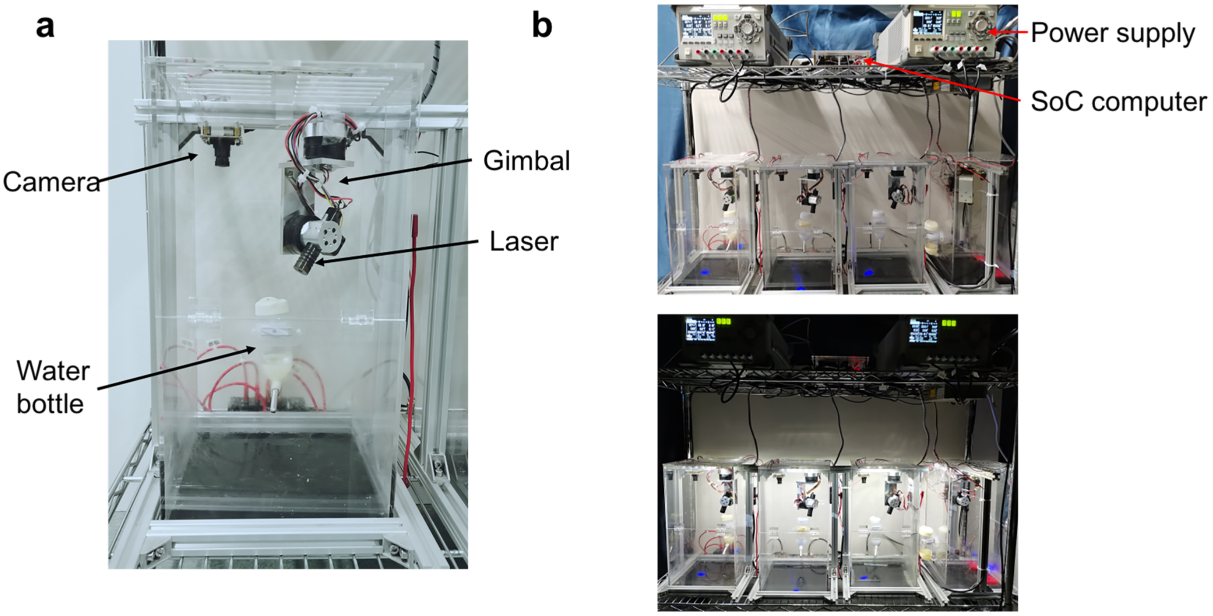

Supplementary Figure 6. Illustration of portable smart light-controllable (PSLC) device in action.
a. The acrylic cage (with water bottle) that equipped with light control hardware. **b.** The entire set of PSLC device, with two power supplies and a microcomputer (upper panel), PSLC device in working condition (lower panel, without mice).

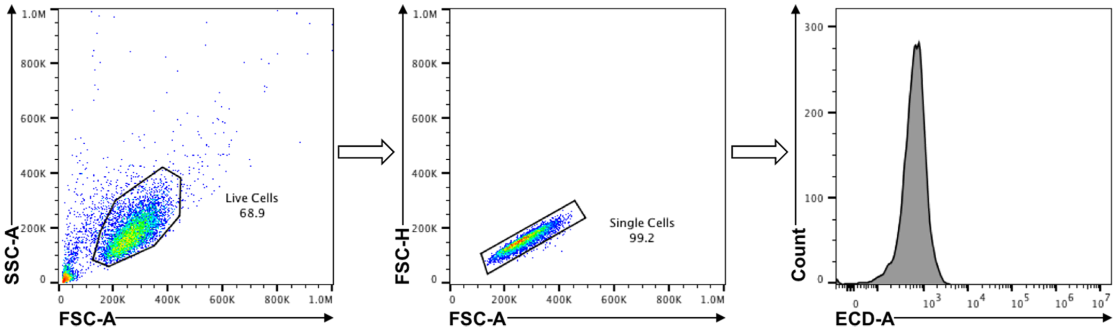

Supplementary Figure 7. Gating strategy for analyzing flow cytometry data.
The stably-transfected P815-M-ILs cells were used for in vitro experiments. After illumination, P815-mRuby-ILs cells were collected from the 24-well plate, filtered through a 40 µm cell strainer to remove clumps, and analyzed with a Beckman Cytoflex S cytometer. The total events are set to 10000/sample. After that, the data was exported and analyzed with FlowJo software. The live cells (68.9% of total events) were captured by forward and side scatter gating (FSC-A and SSC-A), to remove the debris and cell fragments. Subsequently, in a plot of the area versus the height measurement (FSC-A and FSC-H), the single cells were gated (99.2% of live cells), and removed doublet or “clump” cells. Then, the median fluorescent intensity of reporter (mRuby, ECD channel) was calculated from the subset of single cells. Because the red fluorescent protein (mRuby) is a light inducible reporter in P815-mRuby-ILs cells.

Supplementary Table

Supplementary Table 1. Primer sequence for qPCR.

Gene	Forward primers (5'-3')	Reverse primers (5'-3')
Gapdh	CGACTTCAACAGCAACTCCCACTCTTCC	TGGGTGGTCCAGGGTTTCTTACTCCTT
Ifng	CGGCACAGTCATTGAAAGCCTA	GTTGCTGATGGCCTGATTGTC
Cxcl10	ATCATCCCTGCGAGCCTATCCT	GACCTTTTTTGGCTAAACGCTTTC
