# Supplementary material for: Reshaping tumor microenvironment by regulating local cytokines expression with a portable smart blue-light controlled device
Source: Commun Biol. 2024 Jul 29;7:916. doi: 10.1038/s42003-024-06566-y (PMC11289142; doi:10.1038/s42003-024-06566-y)
Supplement: Supplementary file 2 — Description of Additional Supplementary Files [file 42003_2024_6566_MOESM2_ESM.pdf]

Description of Additional Supplementary Files

File name: Supplementary Data 1.

Description: The source data behind the graphs in the main paper.

File name: Supplementary Data 2.

Description: The source data behind the graphs in the supplementary information.

File name: Supplementary Movie 1 (Tracking).

Description: The real-time tracking of target area of mice with PSLC device.

File name: Supplementary Movie 2 (Illuminating).

Description: The real-time illuminating of target area of mice with PSLC device.
