# Supplementary material for: Reshaping tumor microenvironment by regulating local cytokines expression with a portable smart blue-light controlled device
Source: Commun Biol. 2024 Jul 29;7:916. doi: 10.1038/s42003-024-06566-y (PMC11289142; doi:10.1038/s42003-024-06566-y)
Supplement: Supplementary file 7 — Lasing Reporting Summary [file 42003_2024_6566_MOESM7_ESM.pdf]

nature research

Corresponding author(s): Wei Huang

Lasing Reporting Summary

Nature Research wishes to improve the reproducibility of the work that we publish. This form is intended for publication with all accepted papers reporting claims of lasing and provides structure for consistency and transparency in reporting. Some list items might not apply to an individual manuscript, but all fields must be completed for clarity.

For further information on Nature Research policies, including our data availability policy, see Authors & Referees.

Experimental design

Please check: are the following details reported in the manuscript?

1. Threshold

Plots of device output power versus pump power over a wide range of values indicating a clear threshold

- [ ] Yes
- [x] No

Not relevant

2. Linewidth narrowing

Plots of spectral power density for the emission at pump powers below, around, and above the lasing threshold, indicating a clear linewidth narrowing at threshold

- [ ] Yes
- [x] No

Not relevant

Resolution of the spectrometer used to make spectral measurements

- [ ] Yes
- [x] No

Not relevant

3. Coherent emission

Measurements of the coherence and/or polarization of the emission

- [ ] Yes
- [x] No

Not relevant

4. Beam spatial profile

Image and/or measurement of the spatial shape and profile of the emission, showing a well-defined beam above threshold

- [ ] Yes
- [x] No

Not relevant

5. Operating conditions

Description of the laser and pumping conditions
Continuous-wave, pulsed, temperature of operation

- [x] Yes
- [ ] No

As described in Fig.1

Threshold values provided as density values (e.g. W cm^{-2} or J cm^{-2}) taking into account the area of the device

- [x] Yes
- [ ] No

As described in Fig.3

6. Alternative explanations

Reasoning as to why alternative explanations have been ruled out as responsible for the emission characteristics
e.g. amplified spontaneous, directional scattering; modification of fluorescence spectrum by the cavity

- [ ] Yes
- [x] No

Not relevant

7. Theoretical analysis

Theoretical analysis that ensures that the experimental values measured are realistic and reasonable
e.g. laser threshold, linewidth, cavity gain-loss, efficiency

- [ ] Yes
- [x] No

Not relevant

8. Statistics

Number of devices fabricated and tested

- [x] Yes
- [ ] No

As described in Methods (Design and engineering of portable smart blue-light controlled (PSLC) device).

Statistical analysis of the device performance and lifetime (time to failure)

- [ ] Yes
- [x] No

Not relevant
