# Supplementary material for: Reshaping tumor microenvironment by regulating local cytokines expression with a portable smart blue-light controlled device
Source: Commun Biol. 2024 Jul 29;7:916. doi: 10.1038/s42003-024-06566-y (PMC11289142; doi:10.1038/s42003-024-06566-y)
Supplement: Supplementary file 8 — Reporting summary [file 42003_2024_6566_MOESM8_ESM.pdf]

nature portfolio

Corresponding author(s): Wei Huang

Last updated by author(s): Apr 20, 2024

Reporting Summary

Nature Portfolio wishes to improve the reproducibility of the work that we publish. This form provides structure for consistency and transparency in reporting. For further information on Nature Portfolio policies, see our Editorial Policies and the Editorial Policy Checklist.

Statistics

For all statistical analyses, confirm that the following items are present in the figure legend, table legend, main text, or Methods section.

n/a	Confirmed	
☐	☒	The exact sample size (n) for each experimental group/condition, given as a discrete number and unit of measurement
☐	☒	A statement on whether measurements were taken from distinct samples or whether the same sample was measured repeatedly
☐	☒	The statistical test(s) used AND whether they are one- or two-sided<br>*Only common tests should be described solely by name; describe more complex techniques in the Methods section.*
☒	☐	A description of all covariates tested
☒	☐	A description of any assumptions or corrections, such as tests of normality and adjustment for multiple comparisons
☒	☐	A full description of the statistical parameters including central tendency (e.g. means) or other basic estimates (e.g. regression coefficient) AND variation (e.g. standard deviation) or associated estimates of uncertainty (e.g. confidence intervals)
☐	☒	For null hypothesis testing, the test statistic (e.g. F, t, r) with confidence intervals, effect sizes, degrees of freedom and P value noted<br>*Give P values as exact values whenever suitable.*
☒	☐	For Bayesian analysis, information on the choice of priors and Markov chain Monte Carlo settings
☒	☐	For hierarchical and complex designs, identification of the appropriate level for tests and full reporting of outcomes
☒	☐	Estimates of effect sizes (e.g. Cohen's d, Pearson's r), indicating how they were calculated

Our web collection on statistics for biologists contains articles on many of the points above.

Software and code

Policy information about availability of computer code

Data collection	A description of the software and code has been included in the Methods. Initial processing of imaged data was performed using a package of OpenCV, an open-source tool with basic methods in computer vision, in python language. Subsequently, a color mask is generated by an algorithm to recognize the labeled color of these pictures. The python codes for computer vision tracking and pointing can be found in GitHub: https://github.com/QBioLab/let-there-be-light.
Data analysis	The color mask must undergo a morphology procession, including erosion and dilation. It creates a simple and clear mask image for recognizing the contour of blocks. Then, the centroid of a block is calculated frame-by-frame, creating a continuous track of the target sites in animals. Finally, the centroid is calculated and converted to coordinates, and sent to the gimbal controller to guide the laser diode directivity.

For manuscripts utilizing custom algorithms or software that are central to the research but not yet described in published literature, software must be made available to editors and reviewers. We strongly encourage code deposition in a community repository (e.g. GitHub). See the Nature Portfolio guidelines for submitting code & software for further information.

Data

Policy information about availability of data

All manuscripts must include a data availability statement. This statement should provide the following information, where applicable:

- Accession codes, unique identifiers, or web links for publicly available datasets
- A description of any restrictions on data availability
- For clinical datasets or third party data, please ensure that the statement adheres to our policy

The source data for main graphs can be found in Supplementary Data 1, while the source data for supplementary information can be found in Supplementary Data 2. The raw data of RNA-seq and processed data files have been deposited in the NCBI GEO under the accession-number GSE214760, and the analysis code at https://github.com/QBioLab/Cancer-treatment-project-hui-rong. Other data, such as the real-time tracking and analytical datasets that are too large to be publicly shared, yet they are available from the corresponding author, upon reasonable request.

Research involving human participants, their data, or biological material

Policy information about studies with human participants or human data. See also policy information about sex, gender (identity/presentation), and sexual orientation and race, ethnicity and racism.

Reporting on sex and gender	*Use the terms sex (biological attribute) and gender (shaped by social and cultural circumstances) carefully in order to avoid confusing both terms. Indicate if findings apply to only one sex or gender; describe whether sex and gender were considered in study design; whether sex and/or gender was determined based on self-reporting or assigned and methods used. Provide in the source data disaggregated sex and gender data, where this information has been collected, and if consent has been obtained for sharing of individual-level data; provide overall numbers in this Reporting Summary. Please state if this information has not been collected. Report sex- and gender-based analyses where performed, justify reasons for lack of sex- and gender-based analysis.*
Reporting on race, ethnicity, or other socially relevant groupings	*Please specify the socially constructed or socially relevant categorization variable(s) used in your manuscript and explain why they were used. Please note that such variables should not be used as proxies for other socially constructed/relevant variables (for example, race or ethnicity should not be used as a proxy for socioeconomic status). Provide clear definitions of the relevant terms used, how they were provided (by the participants/respondents, the researchers, or third parties), and the method(s) used to classify people into the different categories (e.g. self-report, census or administrative data, social media data, etc.) Please provide details about how you controlled for confounding variables in your analyses.*
Population characteristics	*Describe the covariate-relevant population characteristics of the human research participants (e.g. age, genotypic information, past and current diagnosis and treatment categories). If you filled out the behavioural & social sciences study design questions and have nothing to add here, write "See above."*
Recruitment	*Describe how participants were recruited. Outline any potential self-selection bias or other biases that may be present and how these are likely to impact results.*
Ethics oversight	*Identify the organization(s) that approved the study protocol.*

Note that full information on the approval of the study protocol must also be provided in the manuscript.

Field-specific reporting

Please select the one below that is the best fit for your research. If you are not sure, read the appropriate sections before making your selection.

☒ Life sciences ☐ Behavioural & social sciences ☐ Ecological, evolutionary & environmental sciences

For a reference copy of the document with all sections, see nature.com/documents/nr-reporting-summary-flat.pdf

Life sciences study design

All studies must disclose on these points even when the disclosure is negative.

Sample size	Through the optical tracking hardware and software combination, up to 4 mice could be individually illuminated simultaneously in this apparatus. Therefore, animal data were collected from 3-4 mice in each group, based on the 3Rs of animal research and the minimal amount of mice required to detect significance. The sample size for the number of injection cells was 100 million cells/mouse, which was based on the literature review and preliminary experiments to establish a quickly tumor-growing model.
Data exclusions	Occasionally, a tumor-bearing mouse mostly stayed at the edge of the cage, and the tumor site was partially blocked. This scenario would interfere with the tumor recognition algorithm. The tracking software records all the raw images, tumor recognition. These records helped identify these mice and exclude them from analysis.
Replication	Each experiment presented in the paper was repeated in multiple animals(between 3 and 4 mice in each group). Although a few animals were

Replication	excluded for analysis due to optical tracking hardware instability (see Data exclusions). The experimental effects were consistent, and all the results in the paper came from the analysis of multiple animals.
Randomization	Animals were assigned randomly to experimental and control groups, and we also established double-tumor bearing mice to compare the difference within the same mouse.
Blinding	Blinding was used during RNA-seq analysis, but not in data collection.

Reporting for specific materials, systems and methods

We require information from authors about some types of materials, experimental systems and methods used in many studies. Here, indicate whether each material, system or method listed is relevant to your study. If you are not sure if a list item applies to your research, read the appropriate section before selecting a response.

Materials & experimental systems

n/a	Involved in the study
☐	☒ Antibodies
☐	☒ Eukaryotic cell lines
☒	☐ Palaeontology and archaeology
☐	☒ Animals and other organisms
☒	☐ Clinical data
☒	☐ Dual use research of concern
☒	☐ Plants

Methods

n/a	Involved in the study
☒	☐ ChIP-seq
☐	☒ Flow cytometry
☒	☐ MRI-based neuroimaging

Antibodies

Antibodies used	1. anti-DEC205 antibody (138201, BioLegend).<br>2. anti-CD3 antibody (clone 17A2, 70-0032-U100, TONBO biosciences).<br>3.anti-CD4 antibody (clone GK 1.5, 14-0041-82, Thermo Fisher Scientific).<br>4. anti-CD8α antibody (clone 53-6.7, 14-0081-82, Thermo Fisher Scientific).
Validation	1. The anti-DEC205 monoclonal antibody was purified by affinity chromatography. It verified the reactivity with the mouse. The application of this antibody in immunoprecipitation (IP), immunohistology staining of frozen tissue sections (IHC-F), and Western blot (WB) has been reported in the literature. Each lot of this antibody is quality control tested by immunofluorescent staining with flow cytometric analysis.<br>2. The 17A2 antibody was purified from tissue culture supernatant via affinity chromatography and reacts with the mouse CD3 complex. The application of this antibody in flow cytometry, IP, and IHC-F has been reported in the literature.<br>3. The GK1.5 monoclonal antibody reacts with the mouse CD4 molecule. It has been tested by flow cytometry analysis of mouse thymocytes and splenocytes. The application reported in the literature includes flow cytometry, IP, and IHC-F.<br>4. The anti-CD8α monoclonal antibody is purified with affinity chromatography and reacts with mouse CD8a molecule. The application reported in the literature includes flow cytometry, IP, and IHC-F. It has been tested by flow cytometric analysis of mouse thymocytes and splenocytes.

Eukaryotic cell lines

Policy information about cell lines and Sex and Gender in Research

Cell line source(s)	The murine mastocytoma cell line P815 was obtained from the Type Culture Collection of the Chinese Academy of Sciences, and performed mycoplasma detection and cell line authentication.
Authentication	The Type Culture Collection of the Chinese Academy of Sciences provided the test report of P815 cell line authentication when we ordered the cell line, which is based on short tandem repeat (STR) profiling.
Mycoplasma contamination	We confirmed all the cell lines tested negative for mycoplasma contamination.
Commonly misidentified lines (See ICLAC register)	No misidentified cell lines

Animals and other research organisms

Policy information about studies involving animals; ARRIVE guidelines recommended for reporting animal research, and Sex and Gender in Research

Laboratory animals	DBA/2 mice, NCG mice, 6-8 weeks old
Wild animals	n/a

Reporting on sex	Both male and female are used in this study.
Field-collected samples	n/a
Ethics oversight	Animal protocols and procedures were approved by the Laboratory Animal Welfare and Ethics Committee, Southern University of Science and Technology (approval number: SUSTC-2019-104).

Note that full information on the approval of the study protocol must also be provided in the manuscript.

Plants

Seed stocks	*Report on the source of all seed stocks or other plant material used. If applicable, state the seed stock centre and catalogue number. If plant specimens were collected from the field, describe the collection location, date and sampling procedures.*
Novel plant genotypes	*Describe the methods by which all novel plant genotypes were produced. This includes those generated by transgenic approaches, gene editing, chemical/radiation-based mutagenesis and hybridization. For transgenic lines, describe the transformation method, the number of independent lines analyzed and the generation upon which experiments were performed. For gene-edited lines, describe the editor used, the endogenous sequence targeted for editing, the targeting guide RNA sequence (if applicable) and how the editor was applied.*
Authentication	*Describe any authentication procedures for each seed stock used or novel genotype generated. Describe any experiments used to assess the effect of a mutation and, where applicable, how potential secondary effects (e.g. second site T-DNA insertions, mosiacism, off-target gene editing) were examined.*

Flow Cytometry

Plots

Confirm that:

☒ The axis labels state the marker and fluorochrome used (e.g. CD4-FITC).

☒ The axis scales are clearly visible. Include numbers along axes only for bottom left plot of group (a 'group' is an analysis of identical markers).

☒ All plots are contour plots with outliers or pseudocolor plots.

☒ A numerical value for number of cells or percentage (with statistics) is provided.

Methodology

Sample preparation	After 48 hours illumination, P815-mRuby-ILs cells were collected from 24-well plate, filtered through a 40 μm cell strainer to remove clumps, and analyzed with a Beckman Cytoflex S cytometer. The total events are set to 10000/sample.
Instrument	Beckman Cytoflex S cytometer (Brea, US)
Software	FlowJo V10 (Ashland, US)
Cell population abundance	We here used a stably-transfected cell line, therefore, all the live single cells were gated for analyzing the mean value of fluorescent protein (mRuby).
Gating strategy	The red fluorescent protein (mRuby) is a light inducible reporter in P815-mRuby-ILs cells, so we only need to gate the live single cells, as shown in Fig.S7.

☒ Tick this box to confirm that a figure exemplifying the gating strategy is provided in the Supplementary Information.
